# Efficacy and safety of vacuum-assisted excision (VAE) of fibroadenomas: experience in a tertiary centre

**DOI:** 10.1007/s11547-023-01684-9

**Published:** 2023-08-02

**Authors:** Serena Carriero, Catherine Depretto, Andrea Cozzi, Gianmarco Della Pepa, Elisa D’Ascoli, Giovanni Irmici, Chiara Tamburrano, Daniela Ballerini, Alice Bonanomi, Gianfranco Paride Scaperrotta

**Affiliations:** 1 Postgraduate School in Radiodiagnostics, Università degli Studi di Milano, Via Festa del Perdono 7, 20122 Milan, Italy; 2 Fondazione IRCCS Istituto Nazionale dei Tumori, Via Giacomo Venezian 1, 20133 Milan, Italy; 3 Imaging Institute of Southern Switzerland (IIMSI), Ente Ospedaliero Cantonale (EOC) Via Tesserete 46, 6900 Lugano, Switzerland

**Keywords:** Breast, Fibroadenoma, US, Vacuum-assisted excision

## Abstract

**Purpose:**

To evaluate the technical success and efficacy rates of US-guided percutaneous vacuum-assisted excision (VAE) of breast fibroadenomas, also assessing procedural complications and long-term patient satisfaction rates.

**Materials and methods:**

The institutional database of a tertiary breast cancer referral centre was retrospectively reviewed to retrieve all women with fibroadenomas who underwent US-guided VAE between May 2011 and September 2019. We subsequently included in this study all fibroadenomas with a maximum diameter of 3 cm at US and an available histological confirmation obtained by core-needle biopsy before VAE. Immediately after VAE, technical success (defined as the correct VAE execution) and the occurrence of procedural complications were evaluated. Imaging follow-up (US ± mammography) after 6, 12, 24 and 36 months was performed to evaluate technical efficacy (defined as the absence of fibroadenoma recurrence at 6-month follow-up). Long-term patient satisfaction was evaluated with telephonic interviews in October 2022.

**Results:**

We retrospectively included 108 women (median age 46 years) with 110 fibroadenomas diagnosed at core-needle biopsy with a median lesion size at US of 12 mm. Technical success was obtained in 110/110 VAEs (100%). Minor procedural complications (haematomas) occurred in 7/110 VAEs (6%), whereas 8/110 patients had a fibroadenoma recurrence at 6-month follow-up, resulting in a 93% technical efficacy (102/110 VAEs). All patients available for telephonic follow-up (104/104, 100%) reported high satisfaction with VAE results.

**Conclusion:**

US-guided VAE is a safe and effective procedure for the excision of fibroadenomas, representing a viable alternative to surgery, with a low complication rate and high patient satisfaction.

## Introduction

Fibroadenomas represent the most common benign breast lesion in adolescents and young women, being identified in 67–94% of all biopsies performed in women under 20 years of age [1–3].

The management of most fibroadenomas is usually conservative and, currently, excision is only indicated for fibroadenomas larger than 3 cm in diameter, for fibroadenomas with increasing size over time, and for multiple or bilateral fibroadenomas [4, 5]. However, some women do not feel comfortable with observation because of psychological and cosmetic reasons, or because fibroadenomas may sometime cause pain or physical discomfort due to the presence of a palpable lump [1, 5]. Therefore, as much as 75% of these patients come to prefer excision rather than long-term observation of their fibroadenoma [6, 7].

While fibroadenomas are nowadays removed by open surgery [7], surgical excision not only presents procedural, organizational, and economic disadvantages, but also entails a common risk of aesthetic damage due to surgical scarring [6]. Thus, the progressive implementation of less costly and less invasive techniques yielding better cosmetic results has been advocated by several groups [5, 6]. In particular, image-guided minimally invasive techniques have achieved encouraging results with potentially low costs and low complication rates for the treatment of benign breast lesions and, in selected cases, even of early-stage breast cancer; these techniques offer several advantages over traditional surgical excision, including smaller incisions and reduced scarring [5, 8].

One of these options is represented by vacuum-assisted excision (VAE), a technique in which a biopsy device that includes a cutting needle and a vacuum suction system is used to target the whole lesion with the goal of complete removal; if this cannot be pursued it is considered appropriate to remove a sample of comparable volume/weight to a diagnostic surgical excision (33 cores with a 9G needle), representing approximately 4 g of tissue [9]. Accumulating evidence supports the use of VAE for the excision of benign breast lesions [3, 4, 6–8, 10], marking it as an effective alternative to surgery for the removal of fibroadenomas [5, 8], with complete excision rates as high as 98.8% [3].

As most published studies on this topic limit their assessment to immediate outcomes of VAE for fibroadenoma removal, we aimed to expand the timeframe of this evaluation. Thus, the two-fold purpose of this study will be to evaluate the immediate technical success of US-guided VAE for the removal of fibroadenomas and its safety (in terms of procedural complications) and also appraise the long-term technical efficacy of VAE by considering recurrence rates at different follow-up times and patient satisfaction with the procedure.

## Materials and methods

### Study design

This retrospective, monocentric study was conducted after approval from the local Institutional Review Board. The institutional database of the Breast Imaging Unit of IRCCS Istituto Nazionale dei Tumori (Milano, Italy) was searched to retrieve all patients who had a 14G core-needle biopsy diagnosis of a fibroadenoma (smaller than 3 cm at diagnostic imaging) between 2011 and 2019 and were subsequently treated with US-guided VAE.

### VAE procedure and follow-up

**Fig. 1:**
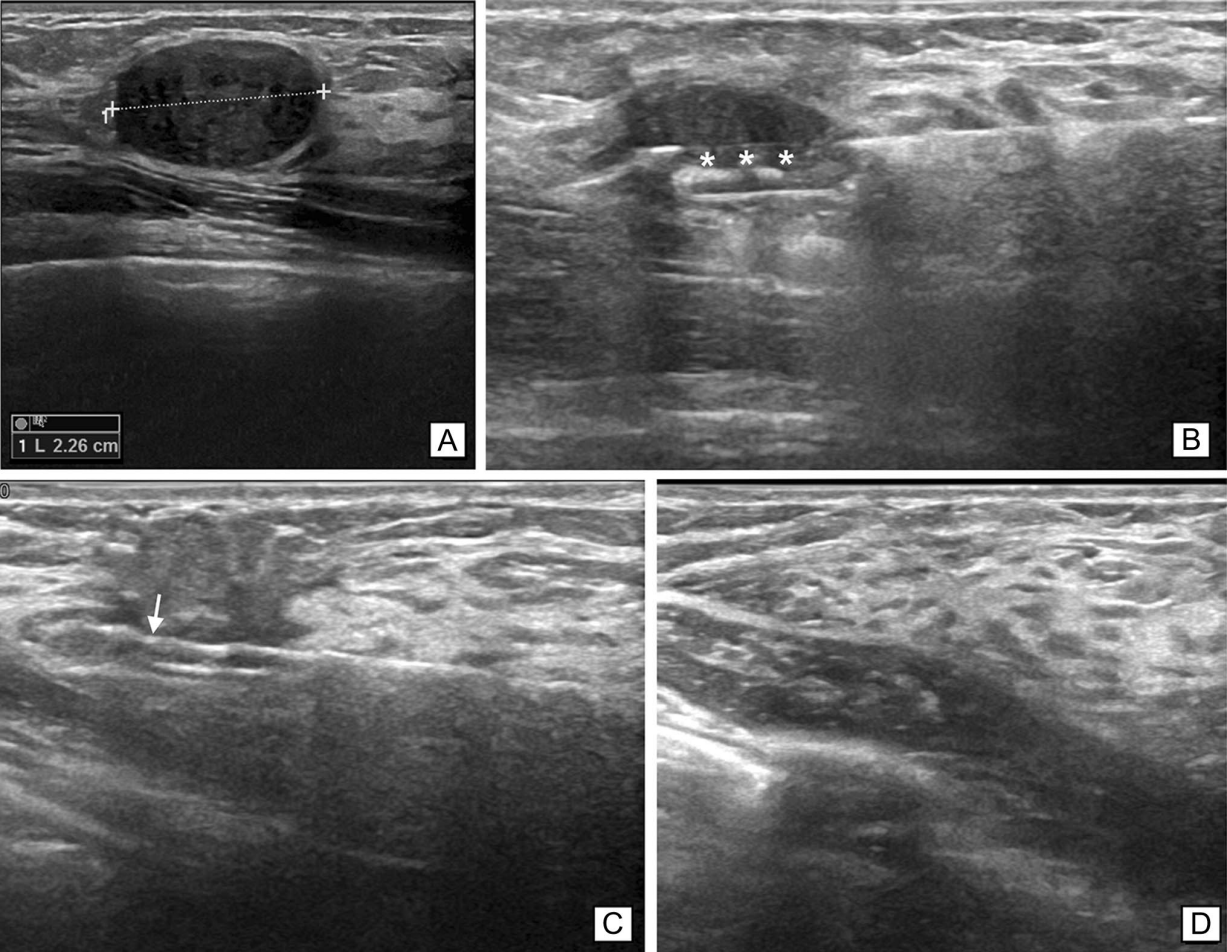
Fibroadenoma successfully treated with US-guided VAE. **A**: Fibroadenoma with a maximum diameter of 2.26 cm in a 26 years old patient, suitable for US-guided VAE. **B**, **C**: Correct positioning of the 9G VAE needle within the lesion during the procedure. In panel **B** the biopsy window (asterisks) is open; in panel **C** the cutting edge (arrow) is closing the biopsy window during suction. **D**: 1-year follow-up showing no residual lesion

In the 2011–2019 timeframe, all VAE procedures were performed by a board-certified breast radiologist with 20 years of experience in interventional breast procedures. After local anaesthesia with 10 ml of 2% Lidocaine, a 9G vacuum-assisted device (ATEC SUROS, Hologic Inc.) was used to perform VAE under US guidance (Logiq E9 US System, GE HealthCare). A 2 mm incision was made, the probe was positioned under the nodule, and the lesion was removed, the collected sample being sent for histopathology. Excision was considered complete when the lesion was no longer visible at US or when a fluid filled cavity or air bubbles were demonstrated by US (panels a, b, and c of Fig. 1). In the vast majority of procedures 4 g of tissue or more were removed in accordance with the standard of the NHS Guidelines for VAE [9]. Nevertheless, in a small number of cases, this was not possible to achieve, especially in sub-centimetric lesions or in small breasts, i.e. where complete removal could be ascertained on US imaging alone and removal of additional tissue was neither necessary nor deemed useful for the patient. After VAE, patients remained under medical observation for 30 min, monitoring the potential emergence of procedural complications, both major (e.g. open bleeding at the excision site) and minor (e.g. formation of haematoma, vasovagal reactions); a tight compression bandage was applied and then removed after 2 days.

Imaging follow-up was carried out at least four times for each patient—at 6 months, 1 year, 2 years, and 3 years, respectively—using only US for patients under 40 years of age (panel d of Fig. 1) and a combined mammography plus US examination for patients aged 40 years or older. In October 2022, we conducted telephone interviews with all enrolled patients to determine their level of satisfaction with the procedure: patients were asked to score their overall satisfaction on a scale from 1 to 5, with “1” denoting extreme dissatisfaction and “5” indicating complete satisfaction, considering the procedure’s comfort as well as their perceived final cosmetic outcome.

### Data analysis

For all included patients, the following endpoints were evaluated: technical success of VAE, procedural complications, technical efficacy of VAE, and fibroadenoma recurrence during follow-up. Technical success was defined as the correct execution of the entire VAE procedure, and technical efficacy was defined as the absence of fibroadenoma recurrence at the 6-month follow-up: keeping a conservative approach, even the visualization of minimal residual at imaging was considered as a fibroadenoma recurrence.

Continuous variables were summarized as mean ± SD or as median and interquartile range (IQR) according to each variable distribution, assessed with the Shapiro–Wilk test. Associations of patient and lesion characteristics (i.e. patients’ age, fibroadenoma size, and fibroadenoma location) with procedural complications and with the presence of recurrence at the 6-month and 3-year follow-up examinations were investigated with the Mann–Whitney *U* test for continuous variables and with the Pearson’s *χ*^2^ test for nominal variables, considering a *p* < 0.05 threshold for statistical significance. All analyses were conducted using STATA (version MP 17.1, Stata Corp).

## Results

Over an 8 year timespan (from May 2011 to September 2019), we included a consecutive series of 108 women (median age 46 years, IQR 39–51 years) with 110 fibroadenomas smaller than 3 cm diagnosed at US-guided core-needle biopsy (2 women had 2 lesions each) who underwent US-guided VAE with a 9G needle. The median lesion size at US was 12 mm (IQR 10–15 mm). As shown in Table 1, 70% (77/110) of all excised lesions were in the outer quadrants of the breasts, the upper-outer quadrant representing the most frequent location (60/110 lesions, 55%). Procedural complications occurred in 7/110 VAEs (6%), all cases being minor complications represented by post-excision immediate hematoma treated with tranexamic acid and antibiotic therapy. No patient had skin injuries as a result of the procedure. The rate of VAE technical success was 100% (110/110). All 108 patients had a follow-up of at least 3 years, whereas 34/108 patients (31%) also had a follow-up of 4 years or more, up to 9 years (5/108 patients, 5%). At short-term follow-up (6 months after VAE) only 8/110 fibroadenomas (7%) presented a recurrence, with a median size of 6 mm (minimum 3 mm, maximum 8 mm, IQR 5–7 mm), with an ensuing 93% rate of technical efficacy (102/110). Of note, all 8 recurrences did not qualify for surgical excision from a clinical point of view, and were managed with follow-up. In 2/8 cases (25%) this recurrence could not be identified at subsequent follow-up examinations at 1, 2, and 3 years, whereas in the other 6/8 cases (75%) the same recurrence could still be identified at all subsequent follow-up examinations (up to 3 years). All remaining lesions with no recurrence after VAE did not present any recurrence at 3-year follow-up or at any further follow-up timepoint.

**Table 1:** Fibroadenoma location

		Side	
		Right breast	Left breast
Fibroadenoma location	Central or areolar	4 (3.6%)	1 (0.9%)
	Upper-outer quadrant	31 (28.2%)	29 (26.4%)
	Upper-inner quadrant	6 (5.5%)	14 (12.7%)
	Lower-outer quadrant	9 (8.2%)	8 (7.3%)
	Lower-inner quadrant	4 (3.6%)	4 (3.6%)

At the time we conducted the satisfaction survey, 4/108 patients (4%) could not be reached; all 104 available patients (100%) expressed high levels of satisfaction (rating between 4 and 5).

As detailed in Table 2, distribution analyses of continuous variables (patients’ age and fibroadenoma size) did not reveal any significant association with procedural complications (*p* ≥ 0.403), with the presence of recurrence at 6-month follow-up (*p* ≥ 0.096), or with the presence of recurrence at 3-year follow-up (*p* ≥ 0.054). Likewise (Table 3), fibroadenoma location according to breast quadrants was not associated with procedural complications (*p* = 0.550), nor with the presence of recurrence after excision (*p* = 0.842) or with the presence of recurrence at 3-year follow-up (*p* = 0.895).

**Table 2:** Association of patients’ age and fibroadenoma size with procedural complications and recurrence

		Patients’ age	*p* value	Fibroadenoma size	*p* value
Occurrence of procedural complications	Yes (7/110 patients)	Median 47 years (IQR 39–49)	0.736	Median 10 mm (IQR 8–13)	0.403
	No (103/110 patients)	Median 46 years (IQR 38–51)		Median 12 mm (IQR 10–15)	
Recurrence at 6-month follow-up	Yes (8/110 patients)	Median 41.5 years (IQR 28–46)	0.096	Median 12.5 mm (IQR 10–20)	0.352
	No (102/110 patients)	Median 46 years (IQR 39–51)		Median 12 mm (IQR 9–15)	
Recurrence at 3-year follow-up	Yes (6/110 patients)	Median 37.5 years (IQR 25–45)	0.054	Median 12.5 mm (IQR 10–20)	0.489
	No (104/110 patients)	Median 46 years (IQR 39–51)		Median 12 mm (IQR 9.5–15)

**Table 3:** Association of fibroadenoma location with procedural complications and recurrence

		Breast quadrants					***p* value**
		Central/Areolar	Lower-inner	Upper-inner	Lower-outer	Upper-outer	
Occurrence of procedural complications	Yes (7/110 patients)	0	1	2	2	2	0.550
	No (103/110 patients)	5	7	18	15	58	
Recurrence at 6-month follow-up	Yes (8/110 patients)	0	1	1	2	4	0.842
	No (102/110 patients)	5	7	19	15	56	
Recurrence at 3-year follow-up	Yes (6/110 patients)	0	1	1	1	3	0.895
	No (104/110 patients)	5	7	19	16	57	

## Discussion

Fibroadenomas are the most common benign lesions of the breast and usually occur in women aged 14–35 years [11], as fibroadenomas are the result of an overgrowth of glandular tissue influenced by hormonal changes that young women undergo at the time of puberty [11]. Most fibroadenomas regress over time—with subsequent calcification and hyalinization—whereas some others may remain stable or experience an increase in diameter: notably, it has been reported that only 0.002% to 0.125% of all fibroadenomas develop into breast cancer [11].

While conservative treatment with follow-up is the standard practice for most fibroadenomas, given their negligible malignant potential, excision is carried out in cases of fibroadenomas larger than 3 cm, in symptomatic fibroadenomas, in cases of fibroadenomas with a documented increase in size, and in the presence of imaging findings of vascularisation and irregular borders [1, 3, 5, 7, 11].

However, according to a previous published study, approximately between 25 and 75% of patients would decide in favour of fibroadenoma excision, were they offered such option [3]. The reasons behind this preference can be traced to patients’ concerns about the potential malignant nature of the lesion (even if reassured by medical professionals), to local minor physical discomfort or pain caused by fibroadenomas, to cosmetic reasons, and to the fact that the required imaging follow-up is seen as a burden, as highlighted by poor patient compliance [3, 5]. While surgery remains the most popular excision option, alternative minimally invasive scarless procedures, such as VAE and ablation, are being progressively implemented [3, 5, 11–13]. The use of VAE for the excision of benign breast lesions has been approved in the United Kingdom by the National Institute of Care and Excellence in 2006, having already obtained an approval in the US by the Food and Drug Administration in 2002 and a subsequent recommendation by the American Society of Breast Surgeons in 2008 [5, 8]. The use of VAE has become increasingly popular in recent years due to its advantages: first, it is much less invasive than traditional surgical techniques, resulting in less pain, less scarring, shorter procedure times, fewer side effects, and shorter recovery times for the patient; second, because of its wider availability and favourable cost–benefit profile [3, 4, 6–8, 10].

To the best of our knowledge, this study presents the first Italian experience on the application of VAE to fibroadenomas. As in previously-published studies outside Italy, we aimed to investigate the efficacy and safety of US-guided VAE for the removal of fibroadenomas in terms of immediate technical success and procedural complications. Moreover, with a long-term follow-up approach, we joined to these aims the appraisal of the efficacy of VAE over time, calculating recurrence rates at different follow-up examinations and evaluating patient satisfaction with telephonic interviews.

In our study population, the rate of technical success of VAE was 100%, in accordance with results from other studies, e.g. the 98.8% success rate reported by Rupa et al. [3, 14, 15]. This high technical success rate can be attributed to the use of large needles (as intrinsic to VAE), the relatively small lesion size, and the high level of experience in breast interventional radiology (more than 20 years) of the operator.

Our complication rate was 6%: no major complications were observed, and all complications were represented by immediate post-excision hematomas, managed conservatively and without any further medical intervention. Our observed complication rate was comparable with a previous study conducted by Salazar et al. [14], who reported a 9% rate of clinically significant hematoma for lesions with a 16–25 mm size. According to our findings and the existing literature, the most frequently reported complication after VAE is hematoma, whereas other complications (e.g. skin injury, pain, and vasovagal reaction) remain rare [5, 14, 16].

At 6-month follow-up, conducted with bilateral US with or without mammography, we observed a recurrence rate of 7% (8/110 patients), with a corresponding 93% technical efficacy: the 7% recurrence rate represents an intermediate figure between the few other available data, i.e. the 5% rate reported by Yao et al. [17] and the 15% rate reported by Buğdaycı et al. [18]. This relative lack of published data about recurrence could be attributed to the short follow-up usually implemented for these patients, as potential recurrences can only be appropriately assessed after the complete resolution of the procedural haematoma. Of note, our study is one of the few to present a longer follow-up time (up to 3 years) for all patients and of more than 4 years for 31% of patients, demonstrating no other case of recurrence aside from the 8 cases that already had a recurrence at the (first) 6-month follow-up. This long-term follow-up also allowed us to investigate patient satisfaction with VAE results. In literature, high overall patient satisfaction rates have been reported [3, 4, 11], as confirmed by the 100% satisfaction rate observed in our study, likely driven by the high rate of technical efficacy and ensuing good cosmetic results: nevertheless, the fact that we did not use a standardized scale and collected information sometimes several years after the procedure may have reduced the internal consistency of this evaluation.

Moreover, we investigated if patients’ age and fibroadenoma size had any association with the occurrence of procedural complications and fibroadenoma recurrence at 6-month and 3-year follow-up, without any evidence of significant associations (Table 2). Finally, fibroadenoma location according to breast quadrants was not associated with procedural complications (*p* = 0.550), nor with the presence of recurrence after excision (*p* = 0.842), or with the presence of recurrence at 3-year follow-up (*p* = 0.895). Conversely, previous studies had suggested that the size of the lesion influenced subsequent hematoma formation [14, 19], as larger lesions might lead to equally larger residual cavities, which may in turn increase the risk of hematoma development [14]. Our strict inclusion criteria, namely the inclusion of fibroadenomas with a maximum diameter at US of 3 cm, represent a likely reason behind the fact that our conclusions on this issue differ from those of other studies, as in a majority of cases these complications were related to large lesions. Of note, a recent study by Wang et al. [20] is in accordance with our results, having reported no statistically significant differences between patients with and without post-procedural hematoma in terms of lesion location and several other characteristics, such as BI-RADS grade, US lesion appearance, vascularity, and patients’ age.

Our study has several limitations. First, its retrospective design and the lack of a control group treated with conventional open excision. The latter limitation was driven by the complexity of creating a surgical control group for the women undergoing VAE—who are already a minority among those diagnosed with fibroadenomas—especially considering the strong current trends towards minimally invasive treatment of these lesions, particularly marked in tertiary referral centres such as our institution. Indeed, the monocentric design of our study and its referral centre setting constitute two other limitations, as they allowed us to include only a relatively small sample size if compared to the high incidence of fibroadenomas and implied a bias of higher-than-average experience of the operator performing VAE, thus potentially limiting the generalizability of our results. A final limitation regards the evaluation of patient satisfaction, as this was assessed only with telephonic interviews—without a multi-item questionnaire and a physician-conducted appraisal of cosmetic results but with a subjective overall evaluation—that in some cases took place up to 3 years after the last imaging follow-up.

In conclusion, US-guided VAE represents a promising approach for the treatment of fibroadenomas, providing a readily available and viable alternative to surgical excision, with high technical efficacy, minimum morbidity, and high patient satisfaction.
